# Participation in Church or Religious Groups and its Association with Health: A National Study of Young Canadians

**DOI:** 10.1007/s10943-013-9726-x

**Published:** 2013-05-15

**Authors:** Valerie Michaelson, Peter Robinson, William Pickett

**Affiliations:** 1 Toronto School of Theology, University of Toronto, Toronto, Canada; 2 Wycliffe College, University of Toronto, Toronto, Canada; 3 Department of Community Health and Epidemiology, Queen’s University, Kingston, Canada; 4 Department of Emergency Medicine, Queen’s University, Kingston, Canada; 5 c/o St. James’ Anglican Church, 10 Union St. W, Kingston, ON K7L 3J9 Canada

**Keywords:** Adolescence, Emotional health, Epidemiology, Health, Pediatrics, Religion, Risk-taking, *Shalom*, Sports and recreation, Spirituality, Theology

## Abstract

The purpose of this study was to determine how participation of young Canadians in a church or religious group correlated with holistic health indicators. Health was viewed in terms of risk and protective behaviors, outward looking prosocial behaviors, and measures of internal feelings, with the composite picture of health connecting to the Hebrew concept of *shalom*. A separate analysis of sports-involved children was used as a comparator. Children involved in religious groups reported lower participation in risk behaviors, higher prosocial behaviors, but poorer levels of the more holistic measures of health. Sports-connected youth reported more positive holistic measures of health and some increases in overt risk-taking. Our findings raise theological and practical issues regarding how the church understands itself and lives out its mission. They suggest an emphasis on teaching about behaviors and morality rather than an understanding of *shalom* that is grounded in the Incarnation and in the deeply integrative nature of the Christian life.

## Preamble

In this population-based and national study, we had a unique opportunity to examine participation of young Canadians in church and other religious groups. Our primary aim was to determine whether such participation related to holistic experiences of health. Here, health was viewed in terms of both specific behaviors and deeper indications of emotional health, with the composite picture of health connecting to the Hebrew concept of *shalom*. These analyses and their interpretations were rooted in the Christian commitment of the authors; however, it is hoped that these findings and this discussion will be of interest to a multi-faith audience.

### Theoretical Underpinnings: A Brief Introduction to the Concept of *shalom*

*Shalom* at its highest is enjoyment in one’s relationships. To dwell in *shalom* is to enjoy living before God, to enjoy living in one’s physical surroundings, to enjoy living with one’s fellows, to enjoy living with oneself (Wolterstorff 1983).

In this quotation, Wolterstorff is describing a definition of wholeness that includes the person, their place in this world, and the matrix of relationships that shape their life. A biblical understanding of *shalom* is reflected in the words “completeness, soundness, well-being, wholeness, peace and health” (Botterweck et al. 2006; Strong 2012). Interestingly, the English word *health* includes similar influences, including the Old English word *haelp* (wholeness) and the Old Norse *helge* (holy or sacred). The Latin word *salva* means “be in good health” and shares its root with the word salvation. The concepts of health, wholeness, and spirituality therefore share very similar roots and are connected intrinsically. These connections are significant as health is considered within various religious contexts, including the Christian tradition.

While often translated simply as peace, *shalom* involves more than a mere absence of hostility within relationships. It includes a dynamic sense of a person flourishing in the context of healthy relationships and also bringing healing, reconciliation, and peace into the troubled and broken relationships around them. It holds together both a sense of awe and delight at the wonders of the created world including the human creature, and a passion to join in God’s redemptive action (Plantinga 1993; Field 2002; Wolterstorff et al. 2004). *Shalom* deepens our understanding of the fullness—or wholeness—of what health can be. It brings with it a picture of health in its broadest, most integrative sense. While there is a clear emphasis on right behavior as a response to right relatedness (e.g., around issues of justice), *shalom* also invites people into a larger story of true belonging characterized by right relationships on every level: with God, with the earth, with community, and with self.

### *Shalom* and the Health of Children in the Christian Church

Within many Christian denominations, a basic theological assumption is that life is meant to be characterized by *shalom*; life is grounded in relationship with a dynamic, loving God who shapes healthy relationships within and outside of the church. By implication then, while the church may not explicitly teach the concept of *shalom* to its children, the Christian church as a body is to be a community of wholeness typified by the concept of *shalom*. Such communities invite people, including children, into a new way of being in the world. At its best, the church has the potential to bring children into a holistic context that is characterized, among other things, by community, forgiveness, and the knowledge of one’s own deep belovedness. If churches or other religious groups are grounded in such relationships, then regular participation of children in these groups should be protective and nurturing for their health. Tangible effects would include reduced engagement in health-compromising risk behaviors, higher levels of engagement in positive “prosocial” behaviors (including participation in behaviors that look beyond oneself and toward others), and improved emotional well-being. While these characteristics in essence are typical of those who are experiencing *shalom* in their lives, what is important in the holistic understanding of health that we are presenting as characteristic of *shalom* is the composite effect of the way these health indicators work together. Our hope is not that church-connected children would not only have higher protective trends around risk-taking and associated behaviors, higher participation in prosocial (or outward-looking) behaviors, or improved emotional well-being in isolation, but that the composite effect of a protective trend in all of these areas would nurture children in a life indicative of the fullness of God’s *shalom.*

Only a scant literature base exists that describes relations between religious group involvement and pediatric health. Exceptions are US-based child studies that have demonstrated connections between religious practices and improved moral behavior as well as lower involvement in problem risk behaviors (Ovwigho and Cole 2010). Other studies report positive associations between church attendance, religious faith and devotion, and better health (Strawbridge et al. 2001; Wallace and Forman 1998). Western Canadian researchers have demonstrated correlations between spirituality (though not religion) and higher levels of child happiness (Wallace 2010), while others have found spirituality to be related to lower levels of depression and lower risks for suicide (Cotton et al. 2005; Cotton et al. 2006; Wallace and Forman 1998). The U.S. *National Study of Youth and Religion* reported that religious teenagers report better health than non-religious teens, particularly with respect to outcomes of lower risk-taking, emotional well-being, quality of relationships, community participation and moral reasoning and behavior (Smith and Denton 2005). Finally, results from *Project Teen Canada* resonate with many of these findings, particularly in the area of religious involvement and lower risk-taking behaviors (Bibby 2009; Bibby and Penner 2010).

While these studies are important, their findings also point to important gaps in the existing theological and health literatures for young people. In particular, this pertains to the question of whether religious involvement of children may lead to a more holistic sense of health, a question that is central to the concept of *shalom.*

### Our Research Opportunity

In response to the identified research gaps, we developed a new collaboration between Canadian theological and health researchers to explore relations between religious participation and health. Our study was national in scope and based on the 2009–2010 Health Behaviour in School-Aged Children (HBSC) study. HBSC was conducted to examine the life experiences and health of younger adolescents aged 11–15 years; 26,078 adolescents in and around that age range from 436 Canadian schools participated in this survey cycle. The survey instrument included a module describing involvement of young people in various groups, with one option being “church or religious groups.” It also requested that participating children report on engagement in various risk-taking behaviors, positive “prosocial” behaviors (which included many outward-looking behaviors such as helping others and sharing), and specific feelings and emotions, both negative and positive.

We hypothesized that if church and religious groups were effective in nurturing health among children, that children who reported engagement in church or religious activities would report lives that are more consistent with our theme of *shalom*. This would include fewer destructive risk-taking behaviors, more prosocial behaviors, and improved levels of the more holistic indicators of emotional health. As the benefits of participation in sports to both physical and emotional health are well known (Pate et al. 1996, 2000; Steptoe and Butler 1996), we also compared findings observed for church-connected children with those observed for children who reported involvement in sports clubs or teams. We did this in order to see whether any positive potential effects of church group membership on the health of children simply mirrored those known to exist for other, more secular groups. If that did occur, one interpretation would be that any positive health benefits could be attributed to membership in a group generally, as opposed to specific membership in a church or religious group.

In the end, our hope was that our collaboration and study would provide deeper insights into how the church fulfills its mandates of nurturing children by way of its ministry. This includes protecting children from the harm of overt risk-taking, encouraging prosocial behaviors, including behaviors that look beyond oneself and to others, and encouraging emotional well-being, each of which is a part of providing an invitation into this sense of *shalom*, or fullness of life. It also provided an opportunity to observe the reality of how the church in Canada as a whole is functioning in its relationship with and ministry to children.

## Methods

*Sample*. The 2009–2010 HBSC was the sixth cycle of this health study conducted in Canada. HBSC in turn is an international study conducted in collaboration with the World Health Organization that aims to increase understanding of health and its determinants in populations of young people in some 43 countries (Currie et al. 2008). It involves written health surveys conducted with students in classroom settings, with a focus on the early adolescent years. Surveys in participating countries are conducted every 4 years following a common protocol. The 2009–2010 Canadian HBSC was conducted from November 2009 to May 2010 and involved participants in eight provinces and the three territories. The national sample was stratified by province/territory, type of school board (public vs. separate), urban–rural geographic status, school population size, and language of instruction (French vs. English). Standardized population weights were generated to account for the oversampling in some jurisdictions and the stratification criteria. Children from private schools, home school situations, native reserves, street youth, incarcerated youth, and youth not providing informed consent were excluded.

*Inclusion criteria* for this analysis were as follows: (1) provision of parental consent (explicit or implicit as per local school board requirements); (2) valid responses to a questionnaire module describing participation in various types of groups; and (3) provision of responses to all core items of analytic interest. There were no specific exclusions.

*Study variables* of specific interest were items describing the following: (1) participation or not in the three key groups of interest; (2) engagement in risk-taking behaviors; (3) outward-looking “pro-social” behaviors; (4) internal feelings, both positive and negative; and (5) sociodemographic variables that were considered as key covariates in our models. These variables have been used in multiple surveys and publications that span the past 20 years, and information on their origins and validity/reliability is documented extensively elsewhere (e.g., Currie et al. 2010). We suggest that it is the composite relationship between the first three types of variables that is related to the theoretical concept of *shalom* and in turn to a holistic understanding of the health of children.

1. *Participation in groups*. In a short series of questions, respondents were asked whether they regularly participated (*yes or no*) in clubs or organizations that were organized around specific activities. Participation in “church or religious groups” was the primary group of interest. Participation in “sports club or team” was selected as a second group, used for comparative purposes.
2. *Risk-taking behaviors*. Each student was asked about their engagement in specific risk behaviors. These included smoking (*ever smoked, daily smoking, and early onset of smoking* defined as prior to age 13); misuse of alcohol (*frequent drunkenness* defined as reporting being drunk >3 times in the past 12 months, *binge drinking* defined as “at least monthly” consumption of 5 or more alcoholic drinks on one occasion for boys, and 4 drinks among girls, *early onset of drinking* defined as prior to age 13); use of drugs (*frequent use of cannabis* defined as 3 or more times during the child’s lifetime, *early onset of cannabis use* defined as prior to age 13, *any use of hard drugs* [any lifetime use of ecstasy, amphetamines, opiates, prescription medications to get high, LSD, methamphetamines, saliva, and other hard drugs]); sexual intercourse (*ever, early onset* prior to age 14); violence (*frequent physical fights* defined as 2 or more in the past 12 months, *bullying others* on 2 or more occasions in the past month); and other risks for physical health (*breakfast skipping* at least 1 weekday per week, *infrequent [*<*once per week] vegetable consumption* and also *fruit consumption, toothbrushing* less than daily, and *relative physical inactivity*, defined as being active for 60 min or <2 days per week).
3. *Outward-looking behaviors*. These included prosocial behaviors that encouraged looking beyond oneself toward others. A series of Likert-like items with response categories ranging from 1 (“definitely not like me”) to 6 (“definitely like me”) were asked to address a variety of positive, prosocial behaviors. These included “I do favors for people,” “I often lend things to people,” “I often help people,” “I often compliment people,” and “I often share things with people.” For each measure, high versus low prosocial behavior was indicated by a score of 4–6 versus 1–3 on the Likert-like scale.
4. *Measures of internal feelings*. Indicators of positive internal feelings were as follows: agreement with the statement “I have confidence in myself” and a score of 9 or more on the 10-point *Cantril ladder*, a standard visual analogue scale used to assess emotional health status and that is depicted in the form of a ladder where 10 indicates “the best possible life” and 1 indicates “the worst possible life” (Public Health Agency of Canada 2009/2010). Indicators of negative internal feelings were also rated using the Likert-like scale described above: “loneliness,” “wishing they were someone else,” “helplessness,” “being sorry for the things I have done,” and “feeling depressed or low.”
5. *Additional covariates* considered as potential confounders or effect modifiers included the following: participant sex (*male or female*), grade level (*6–8, 9–10*), socioeconomic status measured by “how well off do you think your family is?” (5 response options: “very well off” through “not at all well off”), family connectedness indicated by the *number of times per week your family sits down at the table together for dinner/supper (0–7)*, and family structure *(intact family with both mother and father* vs. *other family structure*). We also considered the size of the school community using available divisions of urban–rural geographic status (“Dissemination Area” *{mainly remote communities}*, “Rural” *{all other rural areas}*, “Urban Core,” *and* “Other Urban”).

*Analysis*. We described the study population demographically. Next, we subdivided the national population based upon self-reported membership (*yes or no*) in a church or religious group, then again for self-reported involvement in a sports club or team (*yes or no*), and then in combinations defined by group membership (*none, church or religious group only, sports club or team only, both types of groups in combination*). Groups were compared with respect to engagement in the risk-taking behaviors, the outward-looking “prosocial” behaviors, and the indicators (positive and negative) of internal feelings. Statistical tests for between-group differences in proportion were based upon two-sided *p* values generated from bivariate logistic regression models generated using the SAS Procedure PROC GLIMMIX (SAS Institute, Cary, NC, 2010), applied in order to test for differences while accounting for the clustered sampling design.

We then conducted a series of multi-level logistic regression analyses using the same procedures to study possible associations between group membership and each item on our list of potential health outcomes. We assumed fixed effects but random intercepts for schools. Associations were initially examined in bivariate models, then multiple logistic models that adjusted for sex, grade level, urban–rural geography, socioeconomic status, family structure, and the family connectedness measures. These covariates had been selected as a standard set of control variables following exploratory analyses. Adjusted odds ratios and associated 95 % confidence intervals were again estimated with inflation of standard errors to account for clustering. Interpretively, we looked for general and consistent patterns in the potential effects as opposed to highlighting specific relationships on their own.

*Human subjects*. The HBSC survey holds ethical approval from the General Research Ethics Board at Queen’s University, as well as the Health Canada Research Ethics Board. This particular analysis was also approved by the Social Sciences and Humanities Research Ethics Board at the University of Toronto.

## Results

Demographic characteristics of the study population are described in Table 1. Of the 24,244 (weighted *n*) participants that responded to the church or religious group involvement item, there was a roughly equal split between boys and girls, most were in grades 6–8 (with a roughly equal division in the five grade levels), the majority were from large urban centers and from Central Canada, more reported average or better material wealth than not, and the majority were from families with both a mother and father (intact family structures).

**Table 1:** Reported participation in (1) church or religious groups and (2) sports clubs or teams among young people studied in the 2009–2010 Canadian Health Behaviour in School-Aged Children Study

Characteristic	No.	(%)^a^	% rper week] vegetable consumptioneporting participation^a^	
			Church or religious group	Sports club or team
Total responding to church or religious group item	24,244	(100)	16.4	54.6
By sex				
Boys	11,669	(48.1)	15.4	58.9
Girls	12,575	(51.9)	17.3	50.6
By grade level				
6–8	14,584	(60.2)	17.6	56.2
9–10	9,664	(39.8)	14.6	52.2
By urban–rural geographic status				
Large urban	13,918	(57.4)	15.9	52.2
Other urban	4,074	(16.8)	13.4	55.8
Rural	3,758	(15.5)	17.0	60.0
Dissemination area	2,498	(10.3)	22.9	58.0
By region of Canada				
North	73	(0.3)	17.0	57.8
West	7,743	(32.0)	22.5	56.4
Central	15,376	(63.0)	13.1	53.3
East	1,056	(4.4)	19.2	59.3
By socioeconomic status				
Very well off	5,485	(23.5)	19.1	62.2
Quite well off	7,885	(33.8)	14.8	56.0
Average	7,815	(33.5)	16.2	50.3
Not very well off	1,588	(6.8)	15.9	44.5
Not at all well off	586	(2.5)	22.2	48.8
Missing	888			
By family structure				
Intact family	16,206	(67.8)	17.8	59.0
Not intact family	7,697	(32.2)	13.4	49.1
Missing	346			

^a^Proportions weighted by sampling fractions

Table 1 also summarizes group participation for the two major group activities under study. Overall, 16.4 % of young people reported involvement in a church or religious group, and 54.6 % reported involvement in a sports club or team. For church or religious groups, higher prevalence values were reported for: girls, the lower grades, those from rural or dissemination areas, Western Canada, and those perceiving themselves to be not very well off. For sports, higher reported engagement levels were observed for: boys, the lower grades, Eastern Canada, those perceiving themselves to be more economically advantaged, and those from intact families.

In Table 2, findings from four example covariate models are presented to summarize relationships between the six key demographic covariates used in our models, and reports of four different health indicators: two negative items indicative of risk-taking and therefore the absence of *shalom* (frequent physical fights, loneliness) and two positive indicators indicative of outward-looking behaviors and therefore the presence of *shalom* (do favors for people, help people). These covariates were always included in subsequent modeling of relationships between group involvement and health and are provided for illustrative purposes only. Higher odds of fighting were reported by the following groups of young people: boys, the lower grades, and those from remote areas, non-intact families, families with lower reported material wealth, and families that did not eat meals together as often. The same general pattern was observed for loneliness, except that girls reported loneliness more often than boys. For the positive indicators, higher relative odds were reported for both indicators among girls, those from intact families, those reporting families with higher material health, and those from families that ate meals together more frequently.

**Table 2:** Example covariate models for analyses of group membership and outcomes consistent with the absence or presence of *shalom*

Covariate	Example indicators of the absence of *shalom*				Example indicators of the presence of s*halom*			
	Frequent physical fights		Feel lonely		Do favors for people		Help people	
	OR^a^	(95 % CI)	OR^a^	(95 % CI)	OR^a^	(95 % CI)	OR^a^	(95 % CI)
Sex								
Girls	1.00		1.00		1.00		1.00	
Boys	3.16*	(2.93–3.40)	0.74*	(0.70–0.80)	0.67*	(0.64–0.71)	0.60*	(0.56–0.63)
Grade (per year—range 6–10)	0.90*	(0.87–0.93)	0.99	(0.96–1.02)	0.99	(0.96–1.01)	1.01	(0.98–1.04)
Urban–rural geographic status								
Large urban	1.00		1.00		1.00		1.00	
Other urban	0.88	(0.74–1.06)	1.06	(0.92–1.23)	1.02	(0.88–1.19)	0.89	(0.77–1.02)
Rural	1.20*	(1.00–1.45)	0.93	(0.80–1.08)	1.03	(0.88–1.20)	0.94	(0.81–1.09)
Dissemination area (remote)	1.36*	(1.13–1.62)	1.13	(0.97–1.31)	0.90	(0.78–1.05)	0.87	(0.20–3.71)
Family structure								
Intact	1.00		1.00		1.00		1.00	
Not intact	1.27*	(1.18–1.37)	1.28*	(1.19–1.37)	0.79*	(0.75–0.84)	0.89*	(0.83–0.94)
Material wealth (per unit of increased deprivation—range 1–5)	1.11*	(1.07–1.15)	1.31*	(1.27–1.36)	0.87*	(0.84–0.89)	0.90*	(0.87–0.93)
Family meals together per week (per day—range 0–7)	0.93*	(0.92–0.95)	0.92*	(0.90–0.93)	1.07*	(1.06–1.09)	1.07*	(1.06–1.08)

2009–2010 Canadian Health Behaviour in School-Aged Children Study

* OR estimates are significantly different (*p* < 0.05) from no association (OR of 1.0)

^a^Estimated using multi-level procedures; students nested within schools, and SAS PROC GLIMMIX Procedure

In Table 3, models describing relationships between church or religious group involvement and risk-taking indicative of the absence of *shalom* are summarized. Church or religious group involvement was associated with lower relative odds of: smoking (3 indicators), alcohol misuse (3 indicators), drug use (3 indicators), ever having sexual intercourse, and four of five physical health measures, all indicative of protective effects. Sports group or team involvement was associated with lower relative odds of: smoking (3 indicators), drug use (3 indicators), and five of the five physical health measures. Sports team involvement was associated with higher relative odds of drunkenness and binge drinking, ever having sexual intercourse, and physical fighting.

**Table 3:** Percentage of young people reporting risk-taking behaviors consistent with the absence of *shalom*, by participation in groups

Indicator of risk-taking	Participate in church or religious groups					Participate in sports groups or teams				
	Yes *n* = 3,937 Column %	No *n* = 20,257 Column %	*p* value	Adjusted OR^b^		Yes *n* = 13,230 Column %	No *n* = 11,008 Column %	*p* value	Adjusted OR^b^	
				OR	(95 % CI)				OR	(95 % CI)
Cigarette smoking										
Ever smoked	12.2	20.1	<0.0001	0.81	(0.72–0.91)	15.2	23.0	<0.0001	0.77	(0.71–0.83)
Daily smoking	1.6	3.5	<0.001	0.72	(0.54–0.94)	1.7	4.9	<0.0001	0.49	(0.41–0.58)
Early smoking	4.5	9.1	<0.0001	0.99	(0.84–1.16)	5.7	11.4	<0.0001	0.57	(0.49–0.68)
Alcohol^a^										
Frequent drunkenness	6.8	15.0	<0.0001	0.52	(0.45–0.60)	13.5	13.8	0.14	1.23	(1.13–1.34)
Binge drinking	16.4	27.3	<0.0001	0.62	(0.52–0.73)	26.8	24.8	0.04	1.23	(1.11–1.36)
Early drinking	11.8	18.5	<0.0001	0.69	(0.57–0.83)	16.4	18.8	0.03	0.93	(0.83–1.04)
Drug use^a^										
Frequent cannabis use	9.0	20.4	<0.0001	0.46	(0.37–0.56)	16.8	20.9	<0.0001	0.88	(0.78–0.99)
Early cannabis use	3.0	4.9	0.04	0.77	(0.54–1.09)	3.6	5.8	<0.0001	0.72	(0.58–0.89)
Ever hard drug use	9.7	12.8	0.07	0.90	(0.73–1.11)	10.0	15.0	<0.0001	0.77	(0.67–0.88)
Sexual activity^a^										
Ever had sexual intercourse	16.2	26.7	<0.0001	0.71	(0.59–0.85)	25.0	25.7	0.37	1.18	(1.06–1.32)
Early sexual intercourse	7.3	7.6	0.56	1.18	(0.91–1.52)	6.8	8.4	0.04	0.97	(0.82–1.16)
Violence										
Frequent physical fights	19.1	19.8	0.42	1.03	(0.94–1.14)	19.9	17.7	<0.0001	1.14	(1.06–1.23)
Frequent bullying	14.5	16.2	0.07	0.99	(0.89–1.09)	15.4	16.5	0.25	1.02	(0.94–1.10)
Physical health measures										
Frequent breakfast skipping	19.2	29.3	<0.0001	0.78	(0.71–0.86)	19.6	27.5	<0.0001	0.77	(0.72–0.83)
Infrequent vegetable eating	9.9	13.4	<0.0001	0.73	(0.65–0.83)	10.2	16.1	<0.0001	0.65	(0.60–0.71)
Infrequent fruit consumption	9.1	12.1	<0.0001	0.81	(0.71–0.92)	8.0	15.9	<0.0001	0.50	(0.45–0.54)
Infrequent toothbrushing	5.0	5.2	0.42	1.03	(0.87–1.22)	3.7	6.9	<0.0001	0.58	(0.51–0.65)
Physically inactive	4.2	6.6	<0.0001	0.76	(0.63–0.90)	1.6	18.8	<0.0001	0.15	(0.13–0.17)

2009–2010 Canadian Health Behaviour in School-Aged Children Study

Models were adjusted for the following factors: sex, grade level, urban–rural geographic status, family structure, material wealth (SES), and meals as family

^a^Collected in grades 9 and higher only

^b^Estimated using multi-level procedures; students nested within schools, and SAS PROC GLIMMIX Procedure

Table 4 (top of table) summarizes the results of the modeling of relationships between group membership and factors indicative of the presence of *shalom* in terms of participation in prosocial behaviors that involve looking outward toward others. Participation in church or religious groups was related to higher relative odds of five of the five prosocial behaviors. The same general pattern was observed for participation in sports groups or teams, suggesting that both group involvements were associated with positive outward-looking behavioral tendencies. Findings for the internal feelings indicators (bottom of table) were different. For church or religious group participation, while increases were observed for feelings of confidence and reporting “the best possible life,” there was no statistically significant (*p* < 0.05) relationship between group involvement and the outcomes of loneliness, wishing they were someone else, helplessness, and feeling low or depressed. Increases in the odds of reporting regret (sorry for the things I have done) were observed in those reporting religious affiliations. For sports, young people reporting group involvement reported higher relative odds for the two positive indicators of internal feelings and lower relative odds (all *p* < 0.05) for each of the negative indicators, showing a clear protective effect.

**Table 4:** Percentage of young people reporting behaviors and internal feelings consistent with the concept of *shalom*, by participation in groups

Indicator of *shalom*	Participate in church or religious groups					Participate in sports groups or teams				
	Yes *n* = 3,937 Column %	No *n* = 20,257 Column %	*p* value	Adjusted OR^a^		Yes *n* = 13,230 Column %	No *n* = 11,008 Column %	*p* value	Adjusted OR^a^	
				OR	(95 % CI)				OR	(95 % CI)
Outward-looking behaviors										
Helps people	68.5	61.5	<0.0001	1.34	(1.23–1.45)	66.4	58.2	<0.0001	1.41	(1.34–1.50)
Does favors for others	63.3	59.1	<0.0001	1.25	(1.16–1.35)	63.3	55.5	<0.0001	1.40	(1.32–1.48)
Lends things to people	49.1	43.9	<0.0001	1.29	(1.20–1.39)	47.7	41.1	<0.0001	1.30	(1.23–1.38)
Compliments others	73.6	68.9	<0.0001	1.24	(1.14–1.35)	72.6	66.1	<0.0001	1.41	(1.33–1.50)
Shares things	66.0	60.6	<0.0001	1.24	(1.15–1.35)	64.1	58.4	<0.0001	1.29	(1.22–1.37)
Internal feelings										
Confidence in oneself	75.9	72.1	0.0003	1.13	(1.03–1.33)	78.8	65.5	<0.0001	1.73	(1.62–1.84)
Best possible life	31.4	27.2	<0.0001	1.13	(1.03–1.22)	33.1	21.6	<0.0001	1.53	(1.44–1.63)
Loneliness	20.7	21.2	0.55	1.02	(0.93–1.12)	16.7	26.4	<0.0001	0.63	(0.59–0.67)
Wishes they were someone else	27.3	27.1	0.46	1.08	(0.99–1.18)	24.0	30.9	<0.0001	0.80	(0.75–0.85)
Helplessness	20.3	19.3	0.71	1.08	(0.98–1.18)	16.7	22.7	<0.0001	0.77	(0.72–0.83)
Sorry for the things I have done	54.8	47.9	<0.0001	1.21	(1.12–1.30)	47.9	50.5	<0.0001	0.87	(0.82–0.91)
Feeling depressed or low	15.2	15.8	0.96	1.02	(0.92–1.13)	11.6	20.6	<0.0001	0.58	(0.53–0.62)

2009–2010 Canadian Health Behaviour in School-Aged Children Study

Models were adjusted for the following factors: sex, grade level, urban–rural geographic status, family structure, material wealth (SES), and meals as family

^a^Estimated using multi-level procedures; students nested within schools, and SAS PROC GLIMMIX Procedure

The final analysis (Table 5) summarizes the prevalence of each of the health indicators among young people who reported combinations of group involvement (neither group, sports only, church or religious group only, and both groups). Findings for the “sports only” and “church or religious only” groups are fairly consistent with those patterns reported earlier in Tables 3 and 4, with strong evidence that church or religious group involvement is protective for some common risk behaviors (smoking, drinking, cannabis use, sexual intercourse) and the outward-looking prosocial behaviors (e.g., helping and sharing), but a risk factor for some of the physical health measures (e.g., toothbrushing, physical inactivity). Church or religious group vs. sports involvement was also related to deficits in internal feelings (e.g., confidence in oneself and feelings about the best possible life) but also each of the negative indicators of internal feelings (e.g., loneliness, wishing they were someone else).

**Table 5:** Percentage of young people reporting behaviors and internal feelings consistent with the presence then absence of *shalom*, by participation in church and sports (alone and in combination)

Indicator of *shalom*	Participation in groups				Modeled effects			
	Neither *n* = 9,722 Column %	Sports only *n* = 10,518 Column %	Church only *n* = 1,262 Column %	Church + sports *n* = 2,712 Column %	Church only versus sports only		Church + sports versus church only	
					OR^a^	(95 % CI)	OR^a^	(95 % CI)
Cigarette smoking								
Ever smoked	24.2	16.2	13.7	11.4	0.88*	(0.87–0.90)	0.95	(0.75–1.20)
Daily smoking	5.4	1.8	1.7	1.6	1.03	(0.64–1.67)	0.99	(0.56–1.75)
Early smoking	12.1	6.0	4.9	4.3	0.91	(0.56–1.48)	1.18	(0.63–2.20)
Alcohol^a^								
Frequent drunkenness	14.9	15.0	5.5	7.4	0.31*	(0.23–0.41)	1.71*	(1.23–2.39)
Binge drinking	26.3	28.4	10.3	19.6	0.31*	(0.22–0.43)	2.46*	(1.67–3.65)
Early drinking	19.5	17.5	12.6	11.4	0.71*	(0.52–0.97)	1.05	(0.71–1.56)
Drug use^a^								
Frequent cannabis use	22.3	18.4	8.3	9.4	0.42*	(0.29–0.60)	1.43	(0.91–2.26)
Early cannabis use	6.2	3.7	2.3	3.3	0.83	(0.43–1.60)	1.95	(0.82–4.67)
Ever hard drug use	15.5	10.2	10.8	9.1	0.97	(0.69–1.36)	0.89	(0.58–1.36)
Sexual activity^a^								
Ever had sexual intercourse	27.0	26.5	13.0	17.8	0.45*	(0.32–0.62)	1.51*	(1.02–2.24)
Early sexual intercourse	8.8	6.5	5.1	8.5	0.71	(0.43–1.19)	2.03*	(1.10–3.73)
Violence								
Frequent physical fights	17.9	19.7	16.0	20.5	0.85	(0.71–1.01)	1.25*	(1.02–1.52)
Frequent bullying	16.8	15.6	14.6	14.5	0.96	(0.80–1.15)	1.04	(0.84–1.27)
Physical health measures								
Frequent breakfast skipping	28.3	19.9	20.6	18.5	0.85*	(0.72–0.99)	1.10	(0.91–1.32)
Infrequent vegetable eating	16.6	10.5	12.0	8.9	1.11	(0.90–1.36)	0.79	(0.62–1.01)
Infrequent fruit consumption	16.3	8.1	12.5	7.6	1.64*	(1.35–2.00)	0.61*	(0.48–0.78)
Infrequent toothbrushing	7.0	3.6	6.2	4.4	1.74*	(1.33–2.29)	0.68*	(0.49–0.93)
Physically inactive	12.2	1.5	8.0	2.1	5.89*	(4.46–7.79)	0.22*	(0.16–0.32)
Outward-looking behaviors								
Helps people	57.3	65.4	64.5	70.3	0.92	(0.80–1.05)	1.31*	(1.12–1.53)
Does favors for others	54.9	62.9	59.7	65.0	0.89	(0.78–1.01)	1.26*	(1.08–1.46)
Lends things to people	40.4	47.1	47.4	50.0	1.07	(0.94–1.21)	1.09	(0.94–1.26)
Compliments others	65.4	72.0	71.2	74.7	0.87*	(0.75–1.00)	1.33*	(1.13–1.58)
Shares things	57.8	63.2	63.0	67.4	0.92	(0.81–1.05)	1.28*	(1.10–1.49)
Internal feelings								
Confidence in oneself	65.1	78.7	68.1	79.5	0.65*	(0.57–0.75)	1.58*	(1.33–1.87)
Best possible life	21.1	32.8	24.5	34.7	0.73*	(0.63–0.85)	1.45*	(1.23–1.72)
Loneliness	26.5	16.3	25.9	18.3	1.79*	(1.55–2.08)	0.67*	(0.57–0.80)
Wishes they were someone else	31.0	23.6	30.9	25.6	1.31*	(1.14–1.51)	0.85*	(0.72–0.99)
Helplessness	23.0	15.8	20.7	20.2	1.17*	(1.00–1.38)	1.14	(0.95–1.38)
Sorry for the things I have done	49.4	46.5	58.3	53.1	1.51*	(1.33–1.72)	0.80*	(0.69–0.92)
Feeling depressed or low	20.7	11.2	19.9	13.0	1.71*	(1.54–2.03)	0.80*	(0.54–0.99)

2009–2010 Canadian Health Behaviour in School-Aged Children Study

Models were adjusted for the following factors: sex, grade level, urban–rural geographic status, family structure, material wealth (SES), and meals as family

* OR estimates are significantly different (*p* < 0.05) from no association (OR of 1.0)

^a^Estimated using multi-level procedures; students nested within schools, and SAS PROC GLIMMIX Procedure

However, in addition, analysis of Table 5 also looks at the potential influence on health of adding sports involvement to church or religious group participation. Findings suggested that the relative odds of reporting smoking or drug use was not associated with the combined group versus church or religious group participation only, but the combined group reported increases in the odds of drinking, sexual behaviors, and fighting. Relative to the “only church or religious” group, young people reporting involvement in both groups also reported better physical health, more outward-looking behaviors, and increased positive feelings of confidence in oneself and having the “best possible life” as well as decreased odds of loneliness, wishing they were someone else, regret, and depression. Sports involvement, while not protective for some overt risk-taking among church-involved children, also had many beneficial effects in terms of physical health indicators, outward-looking prosocial behaviors, and several negative indicators of the absence of *shalom*.

## Discussion

This national study was conducted to examine participation of younger adolescent Canadians in church or religious groups, in order to see whether children involved in such groups reported patterns in their health that were consistent with the theological concept of *shalom.* The most important findings were that while potential improvements in overt risk-taking behaviors and outward-looking prosocial behaviors were demonstrated in religious/church-connected children, this protective trend disappeared for several indicators of internal feelings related to emotional health, which are an important component of a holistic understanding of the health of children. The protective trend reported for church or religious group children was also most evident for substance use and sexual behaviors, but less evident for other aspects of physical health. With respect to sports-related groups that were used as a comparator, some similar trends were observed with respect to lower reported levels of risk behavior and higher prosocial behaviors. However, in a series of analyses involvement in sports we also demonstrated strong and striking relations with reduced loneliness, helplessness, wishing to be someone else, and depression along with increased confidence in oneself and positive feelings about having the “best possible life.” Thus, involvement in a sports club was uniquely correlated with the higher sense of internal wholeness that was of primary interest to this study, although some risk-taking tendencies were also noted in sports-involved children.

Our observation of positive relationships between religious group involvement and some aspects of health is consistent with those documented historically (Strawbridge et al. 2001; Holder et al. 2010; Pearce et al. 2003; Ovwigho and Cole 2010; George et al. 2000). Findings from the most established study on church involvement and its impact on Canadian children, albeit 15–19-year-olds, mirror our own in the area of religious involvement and lower participation in sexual behavior and drug use (Bibby 2009). Similarly, findings from a large US-based study, the *National Study of Youth and Religion*, suggest that religious teenagers report acting differently than non-religious teens, particularly in areas such as lower involvement in risk behaviors and quality of relations (Smith and Denton 2005). Hence, our findings support the existence of some general social patterns that seem to be consistent between countries and cultures. In contrast, our study findings suggesting negative or nonexistent associations between religious group attendance and some more holistic internal feelings are somewhat novel. Few studies have tested for the existence of such associations in large populations of children. Theologically, such holistic measures of health and their relations with group membership are important as they take into account the way that children experience *shalom* within themselves in their lives.

We believe that our study and our collaboration have several strengths. Our sample of children was contemporary, representative of Canadian children, and sufficiently large to permit meaningful subgroup analyses. It permitted a variety of analyses of health behaviors conducted across grade levels, by sex, and by group involvement. Second, to our best understanding our particular focus on church/religious involvement and this more composite, holistic approach toward health is novel in young adolescents. If these associations are indeed valid, there is potential to intervene with policies, educational practice, and organizational structures. In short, findings from this initial study provide basic guidance for theological discussions about the role of church and religious groups in nurturing wholeness in children.

Our study also is subject to limitations. First, selection bias is a possibility. It is conceivable, for example, that children with certain social characteristics would be more likely to attend church or other religious events. While efforts were made to control for such social factors in the regression analysis, findings may be residually confounded and the observed patterns may in part be attributable to some sort of social selection in who attends religious functions, rather than what the church or other religious group is doing per se. Second, our analysis was also limited by the absence of a direct measure of spirituality. A third potential limitation surrounds the children who are not included in the HBSC sample. While our sampling frame includes Canadian children who attend the publicly funded Public School and Separate School Board Schools, approximately 7 % of Canadian children attend private religious-based schools, academic enrichment schools, alternative private schools, are homeschooled, or do not attend school. While this does mean that children in some religious-based schooling settings are excluded from the survey results, it is unlikely that within our Canadian context, removing this 7 % from the sample group substantially affects the representativeness of our results. This is further evidenced by a subanalysis (data not shown) that we performed solely with the young people from the Separate School Board systems. The latter likely included youth with mainly Catholic, Protestant Christian and other religious youth affiliations. This subanalysis yielded very similar findings to the larger analysis that was presented. Fourth, while our data sample included responses from a broad spectrum of children involved in a church or religious group, from very conservative Christians, to very liberal Christians, to Roman Catholics to religious groups other than Christianity, there were no controls for denominational or theological differences in this study and we were unable to examine trends within these more specific groups. Such analyses would be of obvious importance for future study. Fifth and finally, it is always difficult to attach deep and consistent meaning to key phrases and constructs used in a written survey. We do not know, for example, what words like “go to church” or “have feelings of regret” actually mean to the individual children who answered the survey questions, and whether there is consistency in meaning. Additional insight into such factors would assist interpretively. Analogously, we did not have any measures of the quality of the experience (religious or sports-oriented) of these children, which may be very important in regard to their impact on health.

Self-reported measures obtained by written survey are always subject to some measurement errors. For example, self-reports of substance abuse may be more influenced by cultural norms and group identity than by actual participation in these behaviors. With respect to our own Christian traditions, it was challenging to identify children within HBSC who are actively involved in a church community due to the lack of specificity in our questionnaire item. The HBSC item is not limited to Christianity, and there may be variations in how children perceive their involvement in existing groups and organizations across different faiths. Finally, as this is a cross-sectional study that provides information on children for a “snapshot” of time, it is difficult to establish the temporal sequence between group membership and the health indicators under study. Some caution therefore must be taken in interpreting all associations as causal, and our findings require confirmation in longitudinal analyses. That being said, we do view our analyses as an important starting point in understanding potential relations between church involvement and a whole-person approach to child health.

## Implications for Ministry

Findings from this study raise important questions and concerns with respect to ministry to children, both within and outside of the Christian tradition. These pertain to the role of ministry in the health of children and especially the integrated experience of *shalom* in their lives. While involvement in a church or religious group offers some protection with respect to engagement in risk behaviors and improved, outward-looking prosocial behaviors, one is left to wonder why this trend does not extend into these other areas of life that are vital to a more composite experience of holistic health in children, including a protective trend around internal feelings. Indeed, involvement in sports clubs appeared to have more potential impact on some important positive aspects of holistic health than church involvement. This finding is sobering. In our own religious traditions, given that a central claim of the Christian faith is a promise of wholeness or *shalom*, the absence of positive findings for these emotional indicators that are an integral part of holistic health is cause for concern. Regardless of the positive story that church or religious group involvement offers a protective trend around overt risk-taking behaviors and outward-looking prosocial behaviors, the notable absence of a continued protective trend in areas of physical and emotional health is problematic. It is important to note that the church operates within the confines of a broken world, and in its fullest sense, this promise of *shalom* is an eschatological hope rather than a present reality. While it is futile to idealize the church in the present as an absolute actualization of all of God’s promises, it nevertheless has the potential to be an agent of *shalom,* offering a foretaste in the present of God’s ultimate recreation of all things.

Our findings therefore also raise theological and practical issues regarding how the church understands itself and lives out its mission. It is appropriate that the Church is intentional in its teaching about avoiding common risk behaviors that can be destructive to one’s overall health (emotional, physical, mental, and spiritual). That religiously connected children appear to smoke less, abuse alcohol and drugs less often, and avoid early sexual activity more often than their non-connected peers can be interpreted as a positive finding in terms of the role that the church has taken in nurturing children. However, the fact that this general pattern of protection does not extend to behaviors connected to violence and physical health (e.g., frequent fighting, healthy eating habits, and physical activity) suggests, anecdotally, that both the teaching and culture in the church have focused primarily on a narrow understanding of morality and a shallow understanding of the incarnation. Speculatively, perhaps there is a tendency within the church to focus on morality as the goal rather than the result of the Christian life. In contrast, an understanding of *shalom* involves inviting or better drawing all people, including young people, into a communal and integrative experience of the fullness of life. In that context our analysis was grounded in the theological assumption that good behavior is not an end in itself but a response to a life-giving relationship with a dynamic, living God.

On a more positive note, our findings also suggest that religiously connected children reported more positive outward-looking, prosocial behaviors than non-involved peers. Indeed, looking beyond oneself and also attentiveness to right relatedness on all levels is an important component of *shalom* that is reflected in this sort of behavior. Because this pattern is strong in church-connected children, Christian doctrine and formation may be quite important as a determinant of prosocial behavior. This correlates with much traditional Christian teaching and practice, which are often connected with behaviors such as helping and sharing. This is also true of the teachings of many of the world’s major religious traditions.

Our comparisons of findings for the church or religious group with those from the group involved with a sports club or team were also valuable. Involvement in sports appeared to have many positive relations with overall health, particularly around lower-risk behaviors in the area of physical health and higher-outward-looking prosocial behaviors. Our most striking observation was the high positive correlation between involvement in sports and increased positive perceptions of internal feelings. This could be connected to the children feeling a part of a group with a common purpose. It could also be connected with greater physical activity, a greater sense of accomplishment within a team or social context, and a higher sense of group identity. However, there was very little correlation between sports group involvement and reduced engagement in serious overt risk-taking behaviors, and the drinking, sexual, and substance use behaviors were even higher in the sports-involved group. Thus, while sports involvement clearly nurtures some important components of *shalom*, it is only when combined with the protective effect of church involvement that it offers protection in terms of overt risk-taking behaviors. While sports involvement on its own offers a striking protective trend in many areas that are in keeping with *shalom,* it is when it is combined with church involvement, as seen in Table 5, that the full spectrum of health behaviors indicative of *shalom* are observed.

These findings then beg the following question: Irrespective of the findings around overt risk-taking, what is it about sport club involvement that appears to influence measures about internal feelings that are essential to our composite understanding of health that is lacking in church/religious involvement? This may be connected with the reality that fewer children attend church than are involved with sports, and with smaller groups and less time of contact, there is more limited potential for comparable peer interaction. It is also possible that in the church, while attention has been given to providing a safe and nurturing environment, insufficient attention has been given to challenging children with tasks and issues that offer a sense of teamwork, achievement, and accomplishment.

Our finding that the protective effects of church/religious group participation did not extend into the particular internal feelings (measures of loneliness, wishing they were someone else, feelings of helplessness, and feelings of depression) is striking and problematic. For example, one theological assumption of this study is that the Christian faith is inherently one of community and that central to the notion of *shalom* is reconciliation not only with God but with one another. Our core finding, that children in church are just as lonely as those outside the church, reveals a disparity between the promise of the Christian life and its reality, at least for young people, and moreover suggests that the church may not be as effective as it should be in integrating children into the full community of church life. While there is no way of understanding the quality of the church or religious experience of participants reporting involvement from the measures we have available, it is likely that the youth who self-reported their involvement represent those with a meaningful connection to the group rather than a nominal attendance. Our findings were in keeping with those from other studies (Bibby 2009; Bibby and Penner 2010), which report similar (or slightly higher) numbers of youth who are actively involved in church, with meaningful engagement. This suggests that beyond not influencing feelings of loneliness in those who have nominal attendance, church involvement is failing to provide a meaningful intervention in terms of feelings of loneliness in some of its potentially most involved young people. This could be connected to a potential lack of peer connection for these children within the church context; however, peer connection is only one factor in terms of mediating feelings of loneliness, because the church generally has no shortage of caring, older adults.

These findings issue a challenge to those involved in church leadership to take seriously the church’s mission of being an intergenerational community of God’s people seriously, and to be more intentional (while being conscious of appropriate and safe boundaries) about integrating young people into the life of the church family. Previous research has shown that this approach to church ministry has radically transformed church experiences of young people and that the church indeed has the potential for this sort of meaningful intervention in the lives of its young people (Devries 2004).

The *shalom* central to the Christian faith also takes seriously matters of one’s identity and unique belovedness, and one’s gifts with which to engage the world. That many church-connected children reported an increased sense of “wishing they were someone else,” “feel depressed or low,” and feelings of helplessness again affirms that children in the Christian community are often not experiencing the fullness of the promise of *shalom*. A meaningful connection with a church should equip and empower children to experience improvements in these areas of emotional life.

The measure regret was a bit of an anomaly in terms of its relations as well as their meaning and interpretation. While church-connected children did not report higher prevalence values with respect to the other measures around internal feelings, they did report more heightened feelings of regret. This raises questions around how these children understand regret, how it reflects their theological worldview, and what it looks like in their lives. For example, it is important to understand whether regret is understood to be positive or negative: It is unclear whether feelings of regret can be interpreted as having feelings of guilt, having ownership and accountability for one’s actions, or something different altogether.

Areas of life such as physical health, emotional health, and communal life are fundamental to the concept of *shalom*. Thus, our findings, from our Christian perspective, suggest that the essential mission of the church may have become distorted by disintegrative theologies that offer a moral code to be followed rather than an invitation into the new and holistic way of being in the world offered to us by Jesus. One possible reason for our findings may be a shallow understanding of human physicality and the body that emphasizes the spiritual over the physical and reflects a narrow or distorted understanding of the incarnation. This dualism becomes more significant since in recent years there has a been a renewed emphasis on the importance of the relationship between human beings and the world in which we live. This in turn naturally raises interest in a more holistic approach both to the human body and to overall health. Any suggestions regarding the significance of our findings and their interpretation must take into account a complex and deeper issue for theology. It would be misleading to suggest that the Christian life carries with it the straightforward promise of a better life with a corresponding sense of overall well-being. While the promise of *shalom* remains a central affirmation of the Christian faith, there are a number of qualifiers as to how and in what way that *shalom* is to be realized.

Inherent within the assumption that the incarnation informs a Christian understanding of *shalom* is the understanding that the incarnation resulted in conflict with societal norms and values, conflict which eventually led to the crucifixion and death of Jesus. Jesus himself asserted that to follow him was to take up one’s cross daily. In other words, there are ways in which the Christian life inevitably results in feelings of loneliness, helplessness, and regret. This is perhaps more evident for children who will experience conflict and uncertainty as they work to integrate their relationships in society at large with their relationships in the church. While acknowledging this complexity and seeking to allow for it in the assumptions of this study, we would continue to affirm that the promise of *shalom* is a real promise even though it may not be the promise of a “better life” in the way it is understood culturally.

## Conclusion

This national study investigated the involvement of younger adolescent Canadians in church or religious groups, and relations between this involvement and various aspects of their health. It was based theoretically on the idea that regardless of whether or not the terminology of *shalom* is explicitly taught within a church context, church involvement should lead to fuller and more holistic health, as encompassed in the concept of *shalom*.

The doctrine of the Incarnation teaches that the Christian faith is inherently one that takes seriously not only the physicality of our bodies, but the whole of our human personhood and experience. At its best, the church has the potential to invite children into a holistic experience that is characterized by loving community, generous forgiveness, healthy and life-giving boundaries around risk behaviors, healthy and life-giving outward-looking behaviors toward others, an affirmation of the body, and a deep sense of one’s own unique gifts and belovedness. The lack of correlation between religious connection and greater overall physical, emotional, and relational health suggests an emphasis on teaching about behaviors and morality rather than an understanding of *shalom* that is grounded in the Incarnation. Moreover, the apparent narrow emphasis on behavior as opposed to a more holistic understanding of health does not lead to nurturing children in the deeply integrative nature of the Christian faith, summarized in Jesus’ invitation into the fullness of life.

Combined with the findings of others, this research offers a challenge to church leaders to rethink ministry to children, and to provide concrete suggestions as to how the Christian formation of children can be effected in ways that nurture the whole person. Further, it is a call to the church to investigate approaches to pedagogy that consider all aspects of health. While recognizing limitations of the church as a loci in which *shalom* can be realized, this research suggests that by thoughtfully challenging the current paradigms prominent in children’s ministry, the church has the potential to have a more meaningful and holistic impact on the lives of the children in our communities. The possible implications of these findings to breathe new life into the often-tired structures of the church are enormous.
